# Chemokine and cytokine levels in the lumbar cerebrospinal fluid of preterm infants with post-hemorrhagic hydrocephalus

**DOI:** 10.1186/s12987-017-0083-0

**Published:** 2017-12-12

**Authors:** Gakwaya Habiyaremye, Diego M. Morales, Clinton D. Morgan, James P. McAllister, Travis S. CreveCoeur, Rowland H. Han, Mohamed Gabir, Brandon Baksh, Deanna Mercer, David D. Limbrick

**Affiliations:** 1 Department of Neurological Surgery, Washington University in St. Louis School of Medicine, One Children’s Way, 4S20, St. Louis, MO 63110 USA; 2 Barrow Neurological Institute, 350 West Thomas Road, Phoenix, AZ 85013 USA; 3 Department of Neurological Surgery and Pediatrics, Washington University in St. Louis School of Medicine, One Children’s Way, 4S20, St. Louis, MO 63110 USA

**Keywords:** Cytokines, Chemokines, Post-hemorrhagic, Hydrocephalus, CSF, Cerebrospinal fluid, Preterm, Prematurity

## Abstract

**Background:**

Neuroinflammation has been implicated in the pathophysiology of post-hemorrhagic hydrocephalus (PHH) of prematurity, but no comprehensive analysis of signaling molecules has been performed using human cerebrospinal fluid (CSF).

**Methods:**

Lumbar CSF levels of key cytokines (IL-1α, IL-1β, IL-4, IL-6, IL-8, IL-10, IL-12, TNF-α, TGF-β1, IFN-γ) and chemokines (XCL-1, CCL-2, CCL-3, CCL-19, CXCL-10, CXCL-11, CXCL-12) were measured using conventional and multiplexed Enzyme-linked Immunosorbent Assays and compared between preterm infants with PHH and those with no known neurological injury. The relationships between individual biomarker levels and specific CSF cell counts were examined.

**Results:**

Total protein (TP) CSF levels were elevated in the PHH subjects compared to controls. CSF levels of IL-1α, IL-4, IL-6, IL-12, TNF-α, CCL-3, CCL-19, and CXCL-10 were significantly increased in PHH whereas XCL-1 was significantly decreased in PHH. When normalizing by TP, IL-1α, IL-1β, IL-10, IL-12, CCL-3, and CCL-19 levels were significantly elevated compared to controls, while XCL-1 levels remained significantly decreased. Among those with significantly different levels in both absolute and normalized levels, only absolute CCL-19 levels showed a significant correlation with CSF nucleated cells, neutrophils, and lymphocytes. IL-1β and CXCL-10 also were correlated with total cell count, nucleated cells, red blood cells, and neutrophils.

**Conclusions:**

Neuroinflammation is likely to be an important process in the pathophysiology of PHH. To our knowledge, this is the first study to investigate CSF levels of chemokines in PHH as well as the only one to show XCL-1 selectively decreased in a diseased state. Additionally, CCL-19 was the only analyte studied that showed significant differences between groups and had significant correlation with cell count analysis. The selectivity of CCL-19 and XCL-1 should be further investigated. Future studies will further delineate the role of these cytokines and chemokines in PHH.

## Background

Post-hemorrhagic hydrocephalus (PHH) develops in up to 25% of preterm infants with intraventricular hemorrhage (IVH) and is a leading cause of infant hydrocephalus in North America [1, 2]. While the association between IVH and PHH is well established [3], the pathophysiological mechanisms linking these two conditions remain unclear. Data from experimental studies and limited clinical series have implicated neuroinflammation in the pathogenesis of PHH [4–7]. IVH-related blood or blood breakdown products may trigger inflammatory fibrosis or arachnoiditis with gliosis that may contribute to an imbalance in cerebrospinal fluid (CSF) production, absorption, or transit [4, 8].

A number of reports have detailed changes in the CSF levels of IL-1β, IL-6, IL-8, TNF-α, IFN-γ, TGF-β1, and TGF-β2 in the setting of experimental or human PHH [1, 9–13]. Indeed, there is experimental evidence that inhibition of TGF-β or lysophosphatidic acid may prevent the development of PHH [9, 14]. To date, human studies into the neuroinflammatory basis of PHH have largely targeted select proteins and, to our knowledge, have not considered the role of chemokines. Informed by our previous work in CSF proteomics [15], we decided to take a broad approach to survey neuro-inflammatory processes at play in the CSF of human infants with PHH. Commercially available multiplex assays offer the advantage of simultaneously measuring proteins from multiple pathways involved in inflammatory modulation while using very little CSF volume. In the current study, we used multiplex analyses investigate the CSF levels of key inflammatory cytokines (IL-1α, IL-1β, IL-4, IL-6, IL-8, IL-10, IL-12, TNF-α, TGF-β1, IFN-γ) and chemokines (XCL-1, CCL-2, CCL-3, CCL-19, CXCL-10, CXCL-11, CXCL-12) in the setting of human infant PHH.

These and related proteins have been evaluated in various clinical contexts previously and have been reviewed in detail by Turner et al. 2014 [16]. IL-1α, IL-1β, IL-6, IL-8, TNF-α, and IFN-γ, among other functions, provide pro-inflammatory signaling for bone marrow cell proliferation, IgG production, chemotaxis, phagocyte cell activation, and anti-viral, macrophage activation. IL-10 and IL-12 are anti-inflammatory signaling molecules and inhibit cytokine production and activate natural killer cells. TGF-β1 has been shown to inhibit T and B cell proliferation. Chemokines also function in inflammatory and immunological responses. XCL-1 provides chemotactic activity specific for lymphocytes while contributing to regulatory T cell development. CCL-2, CCL-3, and CCL-19 among other functions serve to recruit monocytes to inflammation sites and regulate proliferation of progenitor cells. CXCL-10, -11, and -12 are chemotactic for monocytes and T-lymphocytes and with the exception of CXCL-11, have been upregulated in post-traumatic brain injury studies [16–23].

As these studies suggest, neuroinflammation is likely to be an important process in the pathophysiology of PHH and its associated neurological injury. In the current study, we used multiplex analyses to broadly investigate proteins involved in inflammatory modulation and their relationship to CSF cell counts. Based on the results presented herein, future studies will delineate the role of specific cytokines and chemokines in the pathophysiology of PHH.

## Methods

### Research subjects

Washington University Human Research Protection Office (WU-HRPO) approval was obtained prior to beginning this study (WU-HRPO #201101887). Research subjects comprised two study groups: control and PHH. Control CSF samples were acquired from 31 infants born ≤ 35 weeks post-menstrual age (PMA) without known neurological injury via lumbar puncture (LP) performed as part of routine sepsis/meningitis evaluation. Final microbiological cultures were verified as sterile in all controls. PHH CSF samples were acquired from infants born ≤ 30 weeks PMA with PHH as described previously via clinically-indicated LP [24]. Prior to LP, all PHH subjects demonstrated progressive increase in occipital-frontal circumference, full fontanel, splaying of the sagittal suture ≥ 2 mm [25] and a frontal-occipital horn ratio (FOR) ≥ 0.55 [26]. Thirteen of the 14 PHH infants included in this report required ventriculo-peritoneal (VP) shunts between 34 and 59 weeks PMA (Table 1).

**Table 1:** Characteristics of study subjects with post-hemorrhagic hydrocephalus

Subject ID	Sex	PMA at birth (weeks)	TP (μg/ml)	PMA at CSF sample (weeks)	Temporizing neurosurgical procedure	PMA at temporizing procedure (weeks)	VP shunt surgery PMA (weeks)
1	M	24.00	2369	26.86	RES	27.00	37.57
2	F	29.57	1745	30.29	RES	31.29	36.57
3	F	24.00	6072	26.86	RES	27.86	34.71
4	M	29.00	4564	31.14	RES	31.57	40.86
5	M	26.00	9596	27.43	RES	28.14	38.14
6	M	28.14	2606	28.71	RES	30.14	37.14
7	F	24.57	1270	28.00	RES	30.57	39.29
8	M	24.71	1869	30.57	RES	34.14	59.29
9	M	25.43	1420	27.57	NA^a^	NA	NA
10	M	25.43	2620	28.71	RES	31.00	53.57
11	M	25.86	1902	27.71	RES	28.14	NA^b^
12	M	25.29	3735	27.71	RES	28.00	40.00
13	F	29.57	2331	30.00	RES	31.29	36.57
14	F	24.00	4300	26.43	RES	27.00	37.57

*PMA* post-menstrual age, *TP* total protein, *CSF* cerebrospinal fluid, *VP* ventriculo-peritoneal, *RES* ventricular reservoir, *NA* not available

^a^Subject 9 expired after withdrawal of care by family

^b^Subject 11 developed a *S. capitis* RES infection 12 weeks after RES implantation and device tapping for cerebrospinal fluid removal (CSF). CSF samples prior to the 12-week sample were sterile on culture. After infection, the RES was removed and replaced with an external ventricular drain, which was later successfully weaned, and no shunt was implanted. Subject 10 underwent endoscopic third ventriculostomy with choroid plexus cauterization prior to VP shunt placement. Subject 1 had a shunt malfunction within 6 months of VP shunt implantation

### Cerebrospinal fluid processing

CSF samples were acquired via LP under sterile conditions for clinical purposes and transferred to the St. Louis Children’s Hospital clinical laboratory. The clinical laboratory performed cell counts of the PHH CSF including total cell count, nucleated cells, and red blood cells (all measured as cells/µL). They also performed a differential analysis including neutrophils, lymphocytes, and monocytes (measured as percentages). The laboratory then stored the samples at – 80 °C. At the time of experimental analysis, samples were thawed and centrifuged at 2500 rpm for 6 min. The supernatant was aliquoted and used for biomarker assays.

### Total protein measurements

The Pierce Bicinchoninic Acid protein assay kit (Thermo Scientific; Waltham, Massachusetts) was used to estimate total protein (TP) concentration in each CSF sample. Serum albumin standards as well as CSF samples were placed in microplate wells in duplicate. After addition of the working reagent, the plate was incubated at 37 °C for 30 min. The plate was then cooled to room temperature and absorbance at 562 nm was measured on a plate reader. Total protein concentrations were determined with a 4-parameter logistic standard curve.

### Chemokine and cytokine analysis

Enzyme-linked Immunosorbent Assays (ELISAs) were used to measure concentrations of TGF-β1 and the chemokine CXCL-12 in both control and PHH CSF samples (R&D systems, catalog # DY240 and DY350 respectively, Minneapolis, MN). Sigma-Aldrich ELISA kits (Sigma, catalog #RAB0073 and RAB0515) were used for the measurement of CCL-3 and XCL-1 concentrations. All ELISA samples were run in duplicate and the absorbance at 450 nm was measured on a Versamax plate reader (Molecular Devices, Sunnyvale, CA). Chemokine and cytokine concentrations were determined using a 4 parameter logistic standard curve. Aushon (Billerica, MA) human cytokine array #2 and human chemokine array #2 multiplexes were used to measure concentrations of IL-1α, IL-1β, IL-4, IL-6, IL-8, IL-10, IL-12, TNF-α, IFN-γ, CCL-2, CCL-19, CXCL-10, and CXCL-11. Prefabricated assays for these analytes were run according to the manufacturer’s instructions. Multiplex samples were also run in duplicate and analyzed on the Aushon Ciraplex^®^ Assays system.

Due to differences in TP between samples, we normalized analyte measurements by dividing absolute levels by their corresponding TP. Where specified, statistical comparisons were conducted using these normalized measurements.

### Statistical analysis

CSF analyte levels were reported as mean ± standard deviation (SD), and mean difference between groups with a 95% confidence interval. Comparisons between control and PHH groups were conducted using two-tailed independent samples *t* tests assuming unequal variances in Prism 5.0 (GraphPad Software, La Jolla, CA). Linear regressions between CSF cell count parameters and absolute levels of PHH CSF cytokines and chemokines were performed using Spearman correlation coefficients in SAS 9.3 (SAS Institute, Cary NC). A predetermined significance level of 0.05 was used for all statistical tests.

## Results

### Subject characteristics

Fourteen preterm infants with PHH (Table 1) were included in this study. Ventricular reservoirs (RES) were placed in all infants except one, where the family opted for withdrawal of care. Of the 13 remaining subjects, all except one required a VP shunt. The subject not requiring a VP shunt developed a *S. capitis* infection 12 weeks after RES implantation (after weeks of sterile cultures) and underwent device removal and replacement with an external ventricular drain, which was later weaned and removed. Subject 10 had endoscopic third ventriculostomy with choroid plexus cauterization prior to VP shunt placement. Subject 1 underwent VP shunt revision within 6 months of VP shunt implantation. The control group presented with a broad array of clinical reasons for LP, but the primary reason was for sepsis diagnosis. For both groups, the CSF sampled cultures were monitored for 3.68 ± 0.13 days and remained negative.

Seven out of the 31 control subjects were female, while 5 out of the 15 PHH subjects were female. The mean birth PMA for the PHH group was 25.75 ± 2.19 weeks and for the control group was 30.12 ± 3.23 weeks (p < 0.0001). The CSF sample PMA was also significantly different between the groups (31.86 ± 2.83 for control, 28.88 ± 2.97 for PHH; p = 0.003). Ventricular size measurements were only available for 7 out of the 31 control subjects due to a lack of cranial images. Of the 7, 3 of them had ventricular sizes too small to be accurately measured and the other 4 had an FOR of 0.40 ± 0.05. PHH subjects demonstrated much higher FOR measurements (0.62 ± 0.07) for imaging performed within 24 h of CSF sample collection (p < 0.0001).

### Cerebrospinal fluid total protein, cytokines and chemokines

**Fig. 1:**
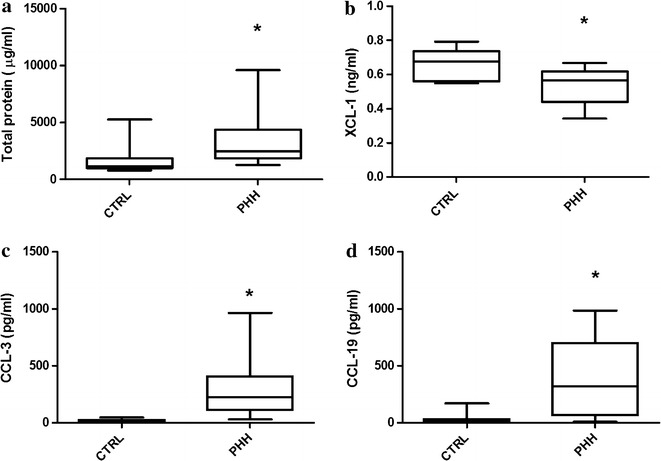
Lumbar cerebrospinal fluid levels of total protein (**a**), XCL-1 (**b**), CCL-3 (**c**), and CCL-19 (**d**) in human preterm infants without (control) or with post-hemorrhagic hydrocephalus. Boxes represent the median with 25th and 75th percentiles and the whiskers show interquartile range multiplied by 1. Levels of Total protein, CCL-3 and CCL-19 were significantly increased in PHH. The levels of XCL-1 were decreased in PHH subjects. *Denotes significance at p ≤ 0.05. *CTRL* control, *PHH* post-hemorrhagic hydrocephalus

CSF TP, cytokine, and chemokine concentrations were measured and compared between control and PHH subjects (Fig. 1a; Tables 2, 3). TP was significantly elevated in PHH versus control (3285 ± 2266 µg/ml vs 1540 ± 985.6 µg/ml, respectively, p = 0.0008). CSF levels of IL-1α, IL-4, IL-6, IL-12, TNF-α, CCL-3, CCL-19, and CXCL-10 were significantly elevated in PHH versus control while IL-1β, IL-8, IL-10, TGFβ1, IFN-γ, CCL-2, CXCL-11, and CXCL-12 were not (Tables 2 and 3). After normalizing by total protein, IL-1α, IL-1β, IL-10, IL-12, CCL-3, and CCL-19 were significantly elevated compared with control, while IL-4, IL-6, IL-8, TNF-α, TGFβ1, IFN-γ, CCL-2, CXCL-10, CXCL-11, and CXCL-12 were not significantly different (Tables 2 and 3). XCL-1 was the only analyte that was significantly decreased in PHH, even when normalized by TP (Table 3). The most robust candidate neuroinflammatory CSF biomarkers—those that retained statistical significance irrespective of normalization by TP levels—included IL-1α (increased), IL-12 (increased), CCL-3 (increased), CCL-19 (increased), and XCL-1 (decreased) (Fig. 1b–d).

**Table 2:** Comparison of cerebrospinal fluid absolute and normalized concentrations of common cytokines in control and post-hemorrhagic hydrocephalus subjects

	Control mean (SD)	PHH mean (SD)	Mean difference	95% confidence interval	p value
Total protein (μg/ml)	1540 (985.6)	3285 (2265.9)	1745.0	2714–775.3	0.0008
IL-1α (pg/ml)	2.385 (3.758)	13.51 (11.03)	11.13	17.67–4.577	0.0019
*Normalized IL*-*1α*	1.58E−7 (2.72E−7)	4.89E−7 (4.56E−7)	3.31E−7	6.40E−7 to 2.31E−8	0.0363
IL-1β (pg/ml)	4.600 (15.05)	79.89 (146.3)	75.29	156.2 to − 5.664	n.s.
*Normalized IL*-*1β*	1.27E−7 (2.93E−7)	1.90E−6 (2.2E−6)	1.78E−6	3.02E−6 to 5.31E−7	0.0072
IL-4 (pg/ml)	0.266 (0.4465)	1.010 (0.5653)	0.7444	− 1.173 to − 0.3164	0.0016
*Normalized IL*-*4*	1.64E−9 (2.53E−9)	4.20E-9 (3.55E−9)	2.56E−9	5.13E−9 to − 1.11E−11	n.s.
IL-6 (pg/ml)	268.2 (667.8)	981.3 (932.6)	713.1	1389–37.32	0.0395
*Normalized IL*-*6*	1.8E−5 (5.6E−5)	4.27E−5 (5.82E−5)	2.49E−5	7.38E−5 to − 2.40E−5	n.s.
IL-8 (pg/ml)	522.5 (528)	1958 (3158)	1436	3205 to − 333.3	n.s.
*Normalized IL*-*8*	4.4E−5 (5.3E−5)	1.04E−4 (1.95E−4)	6.0E−5	1.9E−4 to − 6.7E−5	n.s.
IL-10 (pg/ml)	5.154 (14.59)	128 (274.8)	122.8	274.1 to − 28.38	n.s.
*Normalized IL*-*10*	1.81E−7 (3.13E−7)	2.53E−6 (3.75E−6)	2.35E−6	4.42E−6 to 2.84E−7	0.0277
IL-12 (pg/ml)	1.763 (2.446)	11.58 (9.234)	9.819	15.14 to 4.497	0.0009
*Normalized IL*-*12*	1.3E−7 (2.2E−7)	3.75E−7 (3.12E−7)	2.45E−7	4.70E−7 to 2.043E−8	0.0339
TNF-α (pg/ml)	2.147 (3.337)	9.71 (7.384)	7.566	12.18–2.951	0.0026
*Normalized TNF*-*α*	1.61E−7 (3.0E−7)	3.42E−7 (2.97E−7)	1.81E−7	4.38E−7 to − 7.55E−8	n.s.
TGF-β1 (ng/ml)	0.4504 (0.8607)	1.161 (2.270)	0.7103	2.278 to − 0.857	n.s.
*Normalized TGF*-*β1*	1.9E−5 (2.0E−5)	2.4E−5 (2.1E−5)	4.55E−6	2.02E−5 to − 1.5E−5	n.s.
IFN-γ (pg/ml)	0.6463 (1.552)	1.191 (1.357)	0.5446	1.812 to − 0.7226	n.s.
*Normalized IFN*-*γ*	2.93E−8 (5.65E−8)	3.42E−8 (2.35E−8)	4.91E−9	4.44E−8 to − 3.45E−8	n.s.

*SD* standard deviation, *PHH*: post-hemorrhagic hydrocephalus

**Table 3:** Comparison of cerebrospinal fluid absolute and normalized concentrations of common chemokines in control and post-hemorrhagic hydrocephalus subjects

	Control mean (SD)	PHH mean (SD)	Mean difference	95% confidence interval	p value
XCL-1 (ng/ml)	0.6680 (0.0833)	0.5528 (0.1121)	− 0.1152	− 0.0224 to − 0.2079	0.0176
*Normalized XCL*-*1*	5.4E−5 (1.6E−5)	2.19E−5 (1.1E−5)	− 3.3E−5	− 1.76E−5 to − 4.8E−5	0.0002
CCL-2 (pg/ml)	9436 (5117)	9535 (5089)	99.45	− 4622 to 4821	n.s.
*Normalized CCL*-*2*	7.79E−4 (5.76E−4)	4.16E−4 (4.08E−4)	− 3.62E−4	− 8.47E−4 to 1.21E−4	n.s.
CCL-3 (pg/ml)	16.50 (11.02)	360.0 (327.7)	343.5	537.9–149.1	0.0018
*Normalized CCL*-*3*	0.015 (0.0092)	1.2E−5 (9.9E−6)	− 0.0146	− 0.00658 to − 0.0227	0.0014
CCL-19 (pg/ml)	28.46 (45.15)	410.8 (377.9)	382.4	591.8–173.0	0.0011
*Normalized CCL*-*19*	1.73E−6 (2.14E−6)	1.41E−5(2.1E−5)	1.23E−5	2.36E−5 to 9.96E−7	0.0345
CXCL-10 (pg/ml)	809.6 (776.6)	1480 (742.6)	670	1325–15.09	0.0454
*Normalized CXCL*-*10*	5.9E−5 (5.8E−5)	5.72E−5 (4.42E−5)	− 2.14E−6	4.32E-5 to − 4.75E-5	n.s.
CXCL-11 (pg/ml)	5.162 (10.68)	5.15 (4.58)	− 8.7E−3	7.869 to − 7.886	n.s.
*Normalized CXCL*-*11*	4.08E−7 (1.01E−6)	1.38E−7 (8.01E−8)	− 2.71E−7	4.39E−7 to − 9.80E−7	n.s.
CXCL-12 (ng/ml)	0.5187 (0.5528)	0.9930 (1.0002)	0.4743	1.052 to − 0.1033	n.s.
*Normalized CXCL*-*12*	4.5E−5 (5.3E−5)	1.4E−5 (9.6E−6)	− 3.01E−5	2.01E-5 to − 7.5E−5	n.s.

*SD* standard deviation, *PHH* post-hemorrhagic hydrocephalus

### Cerebrospinal fluid cytokines and chemokines correlation with cell counts

Total cell count measurements for PHH CSF were 72964.86 ± 114192 cells/µL, nucleated cells were 4057.2 ± 13319 cells/µL, and red blood cells were 68907.64 ± 103612 cells/µL. Neutrophils, lymphocytes, and monocytes within the 14 PHH CSF samples were 48.15 ± 33.60, 19.54 ± 21.96, and 21.79 ± 17.32%, respectively. The individual data points for these cell counts were analyzed for a correlation with absolute cytokine and chemokine levels within PHH CSF (Tables 4 and 5). IL-1α correlated with neutrophils with a Spearman r value of 0.73 and a p value of 0.0246. IL-1β correlated with total cell count (r: 0.64; p = 0.0479), red blood cells (r: 0.64; p = 0.0479), neutrophils (r: 0.88; p = 0.0016), and lymphocytes (r: − 0.75; p = 0.0199). IL-6 and IL-10 were significantly correlated with neutrophils (r: 0.67; p = 0.0499 and r: 0.78; p = 0.0075 respectively). TGF-β1 was significantly correlated with nucleated cells (r: 0.83; p = 0.0102). CCL-19 showed significant and strong correlation with nucleated cells (r: 0.71; p = 0.0465), neutrophils (r: 0.78; p = 0.0208), and lymphocytes (r: − 0.74; p = 0.0366). CXCL-10 was significantly correlated with total cell counts (r: 0.63; p = 0.0498), red blood cells (r: 0.63; p = 0.0498), neutrophils (r: 0.88; p = 0.0018), and lymphocytes (r: 0.78; p = 0.0125). CXCL-11 showed correlation with neutrophils (r: 0.67; p = 0.0499), while CXCL-12 showed correlation with lymphocytes (r: − 0.73; p = 0.0396) and monocytes (r: − 0.80; p = 0.0165). XCL-1 and CCL-3 levels did not correlate significantly with any cell counts.

**Table 4:** Post-hemorrhagic hydrocephalus cerebrospinal fluid absolute cytokine Spearman correlations with cell counts

	IL-1α r p value	IL-1β r p value	IL-4 r p value	IL-6 r p value	IL-8 r p value	IL-10 r p value	IL-12 r p value	TNF-α r p value	TGF-β1 r p value	IFN-γ r p value
Total cell count	0.52727 n.s.	0.63636 0.0479	0.29697 n.s.	0.26061 n.s.	0.16387 n.s.	0.49091 n.s.	0.26061 n.s.	0.06667 n.s.	0.69048 n.s.	0.23636 n.s.
Nucleated cells	0.49091 n.s.	0.57576 n.s.	0.15152 n.s.	0.20000 n.s.	0.16387 n.s.	0.50303 n.s.	0.24848 n.s.	0.06667 n.s.	0.83333 0.0102	0.21212 n.s.
Red blood cells	0.52727 n.s.	0.63636 0.0479	0.29697 n.s.	0.26061 n.s.	0.16387 n.s.	0.49091 n.s.	0.26061 n.s.	0.06667 n.s.	0.69048 n.s.	0.23636 n.s.
Neutrophils	0.73333 0.0246	0.88333 0.0016	0.61667 n.s.	0.66667 0.0499	0.12780 n.s.	0.78333 0.0125	0.31667 n.s.	0.05000 n.s.	0.45238 n.s.	0.41667 n.s.
Lymphocytes	− 0.61667 n.s.	− 0.75000 0.0199	− 0.28333 n.s.	− 0.16667 n.s.	− 0.20083 n.s.	− 0.56667 n.s.	− 0.30000 n.s.	− 0.13333 n.s.	− 0.38095 n.s.	− 0.26667 n.s.
Monocytes	− 0.40122 n.s.	− 0.46201 n.s.	− 0.14590 n.s.	− 0.16413 n.s.	0.03424 n.s.	− 0.46201 n.s.	− 0.14590 n.s.	0.09726 n.s.	− 0.35714 n.s.	− 0.19453 n.s.

*r* Spearman correlation coefficient

**Table 5:** Post-hemorrhagic hydrocephalus cerebrospinal fluid absolute chemokine Spearman correlations with cell counts

	XCL-1 r p value	CCL-2 r p value	CCL-3 r p value	CCL-19 r p value	CXCL-10 r p value	CXCL-11 r p value	CXCL-12 r p value
Total cell count	− 0.37143 n.s.	0.09524 n.s.	0.48571 n.s.	0.64286 n.s.	0.63222 0.0498	0.48333 n.s.	0.37126 n.s.
Nucleated cells	− 0.20000 n.s.	0.30952 n.s.	0.08571 n.s.	0.71429 0.0465	0.58967 n.s.	0.63333 n.s.	0.46707 n.s.
Red blood cells	− 0.37143 n.s.	0.09524 n.s.	0.48571 n.s.	0.64286 n.s.	0.63222 0.0498	0.48333 n.s.	0.37126 n.s.
Neutrophils	− 0.25714 n.s.	0.21429 n.s.	0.70000 n.s.	0.78571 0.0208	0.87867 0.0018	0.66667 0.0499	0.70660 n.s.
Lymphocytes	0.31429 n.s.	− 0.09524 n.s.	− 0.54286 n.s.	− 0.73810 0.0366	− 0.78333 0.0125	− 0.58333 n.s.	− 0.73055 0.0396
Monocytes	− 0.25714 n.s.	− 0.30952 n.s.	− 0.55078 n.s.	− 0.54762 n.s.	− 0.46951 n.s.	− 0.53333 n.s.	− 0.80241 0.0165

*r* Spearman correlation coefficient

## Discussion

This study aimed to advance the current understanding of neuroinflammation in PHH by measuring key inflammatory cytokines and chemokines in the CSF. Among 17 CSF biomarkers, 8 were significantly increased (IL-1α, IL-4, IL-6, IL-12, TNF-α, CCL-3, CCL-19, and CXCL-10) and one was significantly decreased (XCL-1) in PHH. Of note, CSF in the clinical setting of PHH contains high levels of protein related to the IVH itself (e.g. albumin); in order to account for any potential effect of CSF protein on CSF biomarker levels, we also report the level of each cytokine and chemokine after normalization by total CSF protein (TP). When normalized by TP, IL-1α, IL-1β, IL-10, IL-12, CCL-3, CCL-19 were elevated, while XCL-1 remained decreased. The most robust candidate CSF biomarkers were significantly altered in PHH irrespective of normalization by TP, which included IL-1α, IL-12, CCL-3, and CCL-19 as increased, and XCL1 as decreased. Of those 5, only absolute levels of CCL-19 correlated with CSF nucleated cells, neutrophils, and lymphocytes, strongly implicating this chemokine in the neuroinflammatory processes of PHH pathophysiology. Neuroinflammatory profile specificity may have important implications in the pathophysiology of PHH and could shape future pharmacological studies in treating PHH.

Neuroinflammation accompanying IVH and PHH is complex and hypothesized to be initiated by blood and its breakdown products in the ventricular system prompting ventriculitis, gliosis and arachnoiditis. Past studies demonstrate that infants with PHH and periventricular white matter injury have increased CSF concentrations of IL-1β, IL-6, IL-8, and TGF-β1 [10, 12, 13, 27, 28]. Although increased levels of TGF-β1 have been implicated in white matter injury in the setting of IVH and PHH, Heep et al. [11] contrarily found no significant difference. A recent review by Szpecht et al. [29] on the role of cytokines in the pathogenesis of IVH suggested an association between IL-1β, IL-6, IL-8, and TNF-α with increased risk of PHH [12, 13, 30].

The current report demonstrates that CSF levels of the cytokines TNF-α, IL-1α, IL-4, IL-6, and IL-12 are significantly increased in PHH. TNF-α and IL-1α findings are consistent with previous studies of their association with acute phase reactions and pro-inflammatory responses to pathogen or tissue injury [31, 32]. Astrocytes, microglia, and neurons typically produce basal levels of TNF-α and maintain homeostasis in normal central nervous system (CNS) physiology; however, just microglia and astrocytes are assumed to be responsible for elevated levels during neuroinflammation [33–37]. IL-1α is constitutively secreted by many cell types, but its expression surges in response to pathogens or brain tissue injury [38]. IL-1α behaves as an upstream signal for multiple proinflammatory cytokines, chemokines, and prostaglandins [31]. Di Paolo et al. demonstrated that IL-1α was the only cytokine to show absolute differences between the two groups with a significant Spearman correlation in the cell count analysis. IL-1α and TNF-α may be associated with acute phase reactions as well as pro-inflammatory states that occur in PHH pathophysiology. Increased levels of TNF-α and IL-6 are also consistent with Savman et al. [12]. Mainly secreted by T-cells, macrophages, and endothelial cells, IL-6 also increases in PHH, which is consistent with previous studies which showed its involvement in neuronal and glial function as well as neuroinflammation pathways in the CNS [39, 40].

IL-4 is a regulatory cytokine that assumes a myriad of immune and non-immune functions. In extravascular tissues, it is involved in alternative activation of macrophages into M2 cells (repair macrophages) and inhibits activation of M1 cells (inflammatory macrophages) resulting in decreased pathological inflammation [41]. It is plausible that increased CSF levels of IL-4 in PHH could be associated with decreased M1 macrophage activity to contain secondary injury from inflammatory cells. Zundler and Neurath [42] describe the main sources of IL-12 as macrophages, monocytes, dendritic cells, granulocytes and B cells; it induces production of IFN-**γ** to foster both innate and adaptive cell-mediated immune responses. Increased levels of IL-12 in PHH could be caused by microglial activation during neuroinflammation to enhance the innate immune response for phagocytosis of extravascular blood, blood-breakdown products, and cell debris resulting from IVH.

Normalization to TP significantly altered the results such that we found selective and significant increases in CSF IL-1α, IL-1β, IL-10, and IL-12 levels in PHH. Furthermore, absolute IL-1β levels significantly correlated with total cell count, red blood cells, neutrophils, and lymphocytes. IL-1β is a potent pro-inflammatory cytokine that initiates and amplifies innate immunity and host responses to microbial and tissue injury [43]. A previous study by Savman et al. [12] showed that IL-1β is significantly elevated in the CSF of premature infants with PHH. Innate responses from phagocytic immune cells, such as macrophages and neutrophils, may be associated with increased IL-1β levels in PHH. We also found a significant correlation between IL-6 and IL-10 levels with neutrophil counts. IL-10 functions in the activation, inhibition, growth, and migration of hematopoietic cells. In the context of tissue injury, whether by infectious or sterile inflammation, IL-10 downregulates and terminates inflammation [44]. Increased IL-10 levels may be associated with down-regulation of neuroinflammation in the setting of IVH or PHH. Contrary to past studies, we did not observe increased TGF-β1 levels in the CSF of PHH subjects [10, 11, 28]. This may have been due to differences between studies such as location of CSF (LP versus ventricular), methods and timing of CSF sample acquisition, or methods of detection. However, there was a significant correlation between absolute TGF-β1 levels and nucleated cells.

To our knowledge, this is the first study investigating CSF chemokine levels in PHH. CSF levels of seven chemokines (XCL-1, CCL-2, CCL-3, CCL-19, CXCL-10, CXCL-11, and CXCL-12) were measured and compared between PHH and control groups. Absolute levels of CCL-3, CCL-19, and CXCL-10 were significantly elevated in the CSF of PHH subjects; CCL-3 and CCL-19 levels remained elevated after normalization with TP. CCL-3, also known as Macrophage Inflammatory Protein 1-α, is primarily secreted by astrocytes, microglia, endothelial cells, and neurons [45–49] and has been found to be upregulated in the CSF of patients after traumatic brain injury [50–53]. CCL-3 is believed to be a potent chemo-attractant of polymorphonuclear leukocytes (PMNLs; neutrophils) in humans and mice [54] and induces peroxide production in PMNLs [55, 56]. In IVH and PHH, reactive oxygen species produced by activated PMNLs may cause local tissue injury, ventriculitis, ependymal layer scarring, and denudation. Chui et al. [48] reported that CCL-3 is a critical early inflammatory chemokine that is greatly upregulated in active Schwann cells and infiltrating macrophages in areas of brain tissue injury. In our CSF samples, CCL-3 is several-fold higher in PHH than controls, thus suggesting that it may have an important role in neuroinflammation.

CCL-19 and CXCL-10 correlated with cell counts, either total, nucleated cells, red blood cells, neutrophils, and lymphocytes. CCL-19, is also known as Macrophage Inflammatory Protein-3β, is involved in immune surveillance of the CNS by lymphocytes. Its ectopic expression may also trigger activation and/or recruitment of infiltrating leukocytes in response to specific offending stimuli [57]. Since CCL-19 is primarily expressed in cerebrovascular endothelium and choroid plexus [57–61], it is readily available to potentially promote neuroinflammation in IVH and PHH. Absolute concentration of CXCL-10 was significantly increased in PHH but there was no difference after normalization by CSF TP. We also found correlations between CXCL10 and total cell count, red blood cells, neutrophils, and lymphocytes, suggesting a possible role in PHH neuroinflammation. CXCL-10 has been reported to be upregulated in post-traumatic brain injury in some studies, but others found absent mRNA expression of CXCL-10 after head injury [21, 50, 62, 63]. Interestingly, both absolute and normalized levels of XCL-1 were decreased in PHH, but absolute levels did not correlate significantly with any cell counts. XCL-1, also known as Lymphotactin, is a chemokine of the –C– class that is expressed by T Cells (Natural Killer Cells, Natural Killer T Cells) in response to pathogenic or injurious stimuli [64]. It is expressed in various infectious and autoimmune diseases, suggesting its predominant role in protective and pathological immune responses [65].

There are a number of limitations in this study. Small sample sizes and inherent clinical heterogeneity were present within both our control and PHH groups. Indeed, non-neurological challenges or conditions could affect CSF levels of neuro-inflammatory markers in both groups. Further, there were also differences between groups in terms of birth and sample PMA (4 and 3 weeks, respectively), raising the possibility of age-dependent variability in CSF flow rate and maturity of arachnoid villi, though in theory, the effect of arachnoid villi maturity would be expected to be minor, since their development is limited until term equivalent age [66–68]. In the PHH group, heterogeneity was inevitably compounded by the complex and dynamic neuro-inflammatory response to IVH and the timing of acquisition of CSF samples, particularly since small molecules, such as those measured in this study, may be metabolized or undergo reuptake into cells in the interstitial fluids and along the CSF pathways. Any combination of these factors could impact cytokine and chemokine levels to such a degree that changes in levels can occur simultaneous with, prior to, or after CSF sampling. Finally, the data for CSF cell types and cell counts reported here were all taken from clinical laboratory reports. Thus, we were reliant on existing institutional clinical laboratory methods and were unable to measure or subtype many cell types (e.g. Th2 cells, CD8 cells) that could provide insight into the immune response itself (protein elaboration, cellular recruitment). These analyses and additional validation studies must be conducted through multi-institutional collaboration and experimental models.

## Conclusion

In summary, the neuroinflammatory processes associated with PHH pathophysiology are complex and remain incompletely understood. In our current study, we measured the levels of CSF cytokines and chemokines in PHH relative to control. CSF levels of IL-1α, IL-4, IL-6, IL-12, TNF-α, CCL-3, CCL-19, CXCL-10, and TP were significantly increased in PHH, whereas XCL-1 was significantly decreased. However, only the cytokines IL-1α and IL-12, and the chemokines CCL-3, CCL-19, and XCL-1 retained their statistical significance for PHH when normalized to CSF TP levels. Furthermore, CCL-19 was the only analyte studied that also had a significant correlation with CSF cell counts. To our knowledge, this is the first study to investigate CSF levels of chemokines in PHH as well as the only one to show that XCL-1 selectively decreased in a diseased state, whether absolute or normalized levels are considered. The selectivity of CCL-19 and XCL-1 should be further investigated. These findings provide novel insights into the neuro-inflammatory processes at play in IVH and PHH, and may help inform future studies of pharmacological treatments for PHH.

## Abbreviations

CCL: CC chemokine ligands
CNS: central nervous system
CSF: cerebrospinal fluid
CXCL: CXC chemokine ligands
ELISA: enzyme-linked immunosorbent assay
FOR: frontal-occipital horn ratio
IFN: interferon gamma
IL: interleukins
IVH: intraventricular hemorrhage
LP: lumbar puncture
PHH: post-hemorrhagic hydrocephalus
PMA: post-menstrual age
PMNL: polymorphonuclear leukocytes
RES: ventricular reservoir
SD: standard deviation
TGF: transforming growth factor
TNF: tumor necrosis factor
TP: total protein
VP: ventriculo-peritoneal
HRPO: Human Research Protection Office
XCL: XC chemokine ligand
